# Projections for obesity, smoking and hypertension based on multiple imputation

**DOI:** 10.1177/14034948211061014

**Published:** 2021-12-14

**Authors:** Jaakko Reinikainen, Tommi Härkänen, Hanna Tolonen

**Affiliations:** Population Health Unit, Finnish Institute for Health and Welfare, Finland

**Keywords:** Prevalence, risk factors, projection, microsimulation, multiple imputation

## Abstract

**Aims:**

Information on the future development of prevalences of risk factors and health indicators is needed to prepare for the forthcoming burden of disease in the population and to allocate resources properly for prevention. We aim to present how multiple imputation can be used flexibly to project future prevalences.

**Methods:**

The proposed approach uses data on repeated cross-sectional surveys from different years. We create future samples with age and sex distributions corresponding to the official national population forecasts. Then, the risk factors are simulated using multiple imputation by chained equations. Finally, the imputations are pooled to obtain the prevalences of interest. Covariates, such as sociodemographic variables as well as their possible interactions and non-linear terms, can be included in the modelling. The future development of these covariates is also projected simultaneously. We apply the procedure to data from five Finnish health examination surveys conducted between 1997 and 2017, and project the prevalences of obesity, smoking and hypertension to 2020 and 2025.

**Results:**

The prevalence of obesity is projected to increase to 24% for both men and women in 2025. The prevalences of hypertension and smoking are expected to continue decreasing, and the differences between men and women are projected to remain so that men will have higher prevalences.

**Conclusions:**

**Simulation of future observations by multiple imputation can be used as a flexible yet relatively easy-to-use projection method.**

## Introduction

Public health monitoring helps to understand the current health state of the population and learn about the past development of health indicators. This information can also be used to foresee the future development of public health and needs for healthcare and disease prevention. However, formal projection methods are needed to evaluate the uncertainty of health projections.

Projection studies investigating different future scenarios have long traditions especially in environmental [1] and economic sciences [2], but they are relatively new in health sciences [3]. The choice of a method for projecting health indicators depends on the type of the indicator and types of available data. Data requirements for simplistic extrapolation methods are small but they cannot take into account the expected changes in background variables which may affect the development of the health indicator.

Time series methods, such as (seasonal) ARIMA [4], have often been used to make projections based on historical data. They usually require many consecutive observations in time so they may not be readily applicable if data from only a few cross-sectional studies are available. If the size of data also varies between the measurement points, these methods in their basic form do not take into account the variability in the amount of information in the time points.

Cancer incidence has been projected using age–period–cohort models [5], which also take into account possible birth cohort effects. Microsimulation-based methods can produce reliable projections, but have, on the other hand, often high requirements for data sources. For example, Kilpi et al. [6] used a refined simulation approach to project obesity trends and obesity-related diseases in alternative scenarios. Their method demanded information on population demographics, obesity, disease incidence, mortality and survival data.

As the aging of populations is reshaping the population structures [7], interest in projections that take into account the expected demographic changes is increasing. Simplistic trend projections do not provide reliable enough information for policy planning. Instead, carefully planned projection studies may give valuable information for policy-makers [8].To be able to allocate preventive actions effectively, projections are needed for different population groups, such as age groups and socioeconomic groups.

Complex projection methods take into account changing population structure and dynamic covariate effects as well as uncertainties related to them, but may be too burdensome to construct for different cases. Thus, there is a need for a reasonable method, which could be applied easily for different purposes.

In this paper, we present a flexible yet relatively easy-to-use method for projecting population-level health indicators. This approach is based on simulation of future observations by multiple imputation and makes use of data on repeated cross-sectional surveys. There is a straightforward way to include necessary risk factors and sociodemographic variables as well as their interactions and non-linear terms in the modelling. We demonstrate the method by projecting the prevalences of obesity, smoking and hypertension to 2020 and 2025 using data from five Finnish health examination surveys conducted between 1997 and 2017.

## Methods

### Data

We use data from five cross-sectional health examination surveys conducted every 5 years between 1997 and 2017 in Finland: the FINRISK 1997–2012 Surveys [9] and the FinHealth 2017 Study [10]. These data are restricted to represent five large geographical areas and the common age range of 25–64 years to be comparable with each other. The main variables, whose prevalences we aim to estimate, are obesity, smoking status and hypertension. Obesity is defined as body mass index greater or equal to 30 calculated from measured height and weight. Smoking status is current smoking reported on a questionnaire. Hypertension is defined as systolic blood pressure being greater or equal to 140 mmHg, or diastolic blood pressure greater or equal to 90 mmHg or the use of medicine for high blood pressure in the past 7 days. Other variables used as covariates were sex, age, survey year, area, marital status and educational level. Age and survey year were considered as continuous variables, area had five and educational level three categories and all others were binary.

Sample sizes and the amount of missingness by sex and survey year are presented in Table I. Proportions of missing values in the three variables of interest were increasing until the year 2012. The participation was always better among women than men.

**Table I.** Sample sizes and amount of missingness in the data from the FINRISK 1997–2012 and the FinHealth 2017 Studies.^a^

		Year				
		1997	2002	2007	2012	2017
Sample size, *N*	Men	5000	4999	4000	4000	1635
	Women	5000	5000	4000	4000	1542
Missing values^b^ in obesity, *N* (%)	Men	1607 (32.1)	2032 (40.6)	1758 (44.0)	1941 (48.5)	787 (48.1)
	Women	1242 (24.8)	1501 (30.0)	1377 (34.4)	1612 (40.3)	591 (38.3)
Missing values^b^ in smoking status, *N* (%)	Men	1631 (32.6)	1764 (35.3)	1598 (40.0)	1741 (43.5)	696 (42.6)
	Women	1260 (25.2)	1249 (25.0)	1192 (29.8)	1357 (33.9)	492 (31.9)
Missing values^b^ in hypertension, *N* (%)	Men	1606 (32.1)	2018 (40.4)	1735 (43.4)	1904 (47.6)	763 (46.7)
	Women	1241 (24.8)	1474 (29.5)	1348 (33.7)	1563 (39.1)	572 (37.1)

a Datasets are restricted to 25–64-year-old people and five large geographical areas to make the studies comparable.

b Missing values among the entire invited samples.

### The projection method

The idea of projecting population-level prevalences of health indicators is to simulate individual-level values in unobserved samples in the future by multiple imputation. That is, we considered the future health examination surveys as missing data. After the imputation, the predicted (imputed) values are pooled to obtain the prevalences of interest.

Data on repeated cross-sectional surveys from different years are used for fitting imputation models. Covariates, such as sociodemographic variables, as well as their interactions and non-linear terms can be included in the imputation models. The future development of these covariates is also projected simultaneously. Usually, there is also missingness in the past surveys due to non-participation and item non-response, which is also the case in our analysis as shown in Table I. These missing data are handled at the same time by multiple imputation. Before the imputation, pseudo-samples with all the values missing are created for those years for which we want to produce projections. Then, age and sex distributions of these future samples are fixed to correspond to the official national population forecasts to take into account the changing population structure. These new data are combined with the observed data to the same data frame. Thus, the rows representing the future samples have fixed values for survey year, age and sex and all the other variables have only missing values before the imputation.

### Multiple imputation

We use multiple imputation by chained equations, also called fully conditional specification [11] to simulate individual-level values in the future. Multiple imputation is a widely used microsimulation method for handling missing data. The central idea is to generate predictions for each missing value multiple times resulting in multiple copies of the original dataset, which contain no missing values. Unlike single imputation techniques where each missing value is imputed only once, multiple imputation takes properly into account the uncertainty related to the imputation process. After the data have been imputed, the parameters of interest are estimated separately with each imputed dataset and then the results are pooled using Rubin’s rules [12] to obtain the final estimates with their standard errors.

Multiple imputation by chained equations is a flexible iterative approach for generating imputations [13]. It can be used for different data types and imputation models, and is especially useful when missingness occurs in more than one variable and the pattern of missingness is not monotonic.

### Application of the method

In the demonstration of the proposed method, mice-package [14] in R software was utilised in multiple imputation. Logistic regression (logreg) and polytomous logistic regression (polyreg) were used as imputation models for binary (obesity, smoking status, hypertension and marital status) and categorical (area and educational level) variables, respectively. Age, sex and survey year had no missing values.

The unobserved samples of the years 2020 and 2025 were created to consist of 10,000 individuals with age and sex distributions matching to the distributions of national population projections in Finland in the same years [15] made in 2018. In the comparison of observed and projected prevalences of 2017, we used the sample size of 3177 for the projections, which was also the size of the observed survey in 2017. In addition to these unobserved future samples, missing values in the past samples were also imputed simultaneously.

The selection of imputation models for the three risk factors of interest was based on the Bayesian information criterion (BIC) [16]. The need for non-linear modelling of continuous variables was tested by using restricted cubic splines [17]. The number of multiply imputed datasets was 50 and the number of iterations in the chained equations imputation was 10. Convergence plots are presented in Supplemental Figure 1. Spearman correlation coefficient was used to examine how well the relationships between the variables remained in fully simulated data.

## Results

The model for smoking, selected using BIC, included interactions of age for obesity, education and marital status. The other variables had only their main effects in the model. The model for obesity included an interaction between year and age and main effects for others. The model for hypertension had interactions of age for marital status and sex and main effects for others. Imputation models for marital status, educational level and geographical area included the main effects of all the other variables. In the model for smoking, a restricted cubic spline with two degrees of freedom was used for survey year, and in all the other imputation models the effect of survey year was considered linear.

**Figure 1.**
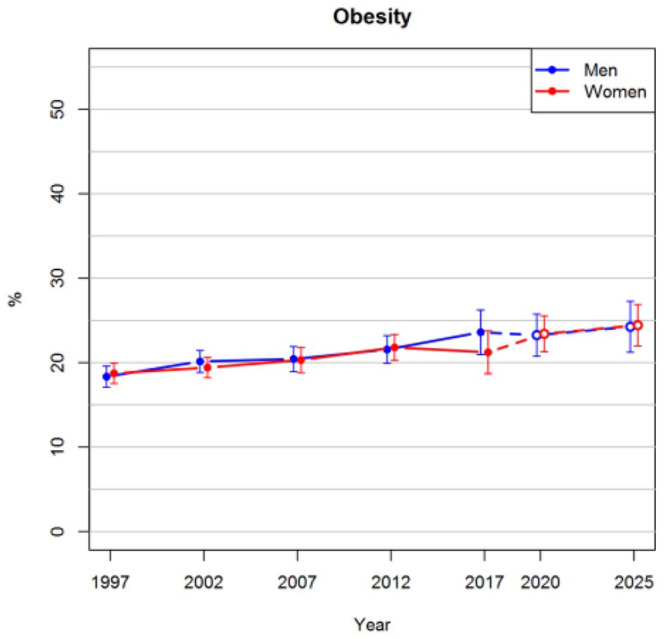
Observed prevalences of obesity for the years 1997–2017 and projections for 2020 and 2025 with 95% confidence intervals.

**Figure 2.**
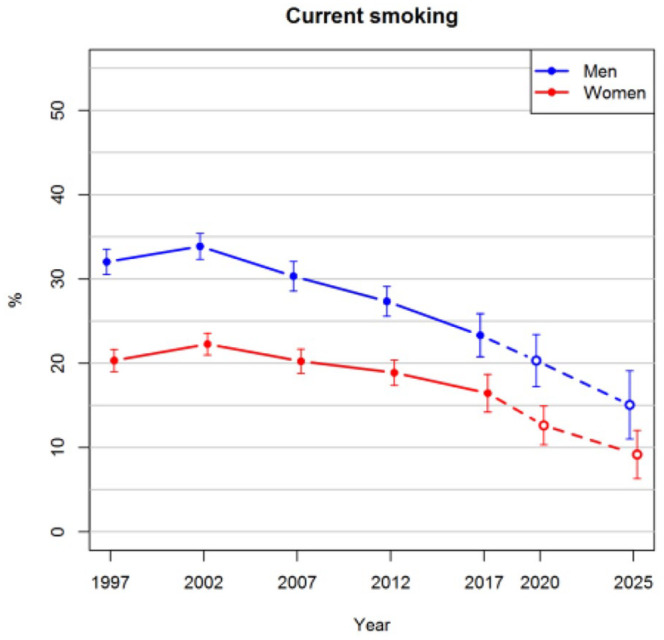
Observed prevalences of current smoking for the years 1997–2017 and projections for 2020 and 2025 with 95% confidence intervals.

**Figure 3.**
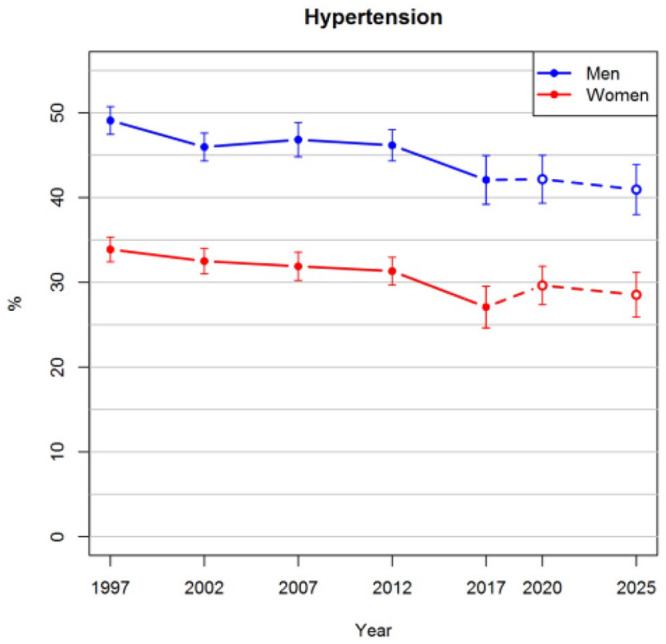
Observed prevalences of hypertension for the years 1997–2017 and projections for 2020 and 2025 with 95% confidence intervals.

Figure 1 shows that the prevalence of obesity in the age range of 25–64 years is projected to increase steadily in the future. The projected prevalences for both men and women in 2025 are 24%. In the observed data, there was not enough evidence that the trends of men and women would have diverged after 2012, and thus an interaction between sex and survey year was not included in the imputation model. The proportion of smokers has been decreasing after 2002 among both men and women, as can be seen in Figure 2. These trends are projected to continue so that approximately 9% of women and 16% of men would be smokers in 2025. The prevalences of hypertension slightly decreased before 2012, after which a greater drop was observed in 2017 (Figure 3). The long-term trend is projected to be gradually decreasing also in the future.

A sensitivity analysis was carried out to demonstrate the importance of careful imputation model selection. Supplemental Figure 2 shows clear differences in projected prevalences of current smoking when the effect of the survey year was considered linear instead of using a spline.

We calculated the correlations between the risk factors and covariates for each year to verify that the structure of the simulated data remained plausible. Although the data for 2020 and 2025 were fully simulated, the relationships between the variables were similar to those in the past surveys (Supplemental Figure 3). For example, the correlation between age and hypertension remained slightly less than 0.4, whereas the correlation between obesity and smoking stayed approximately at zero.

Agreement between observed and projected prevalences was also compared. The prevalences of obesity, smoking and hypertension in 2017 were projected using data from 1997 to 2012 and compared with the observed prevalences of 2017 (Table II). In all cases, the prediction intervals include the observed prevalences except for hypertension among women. In hypertension, there was a drop after 2012 which could not be projected using the data.

**Table II.** Comparison of observed and projected prevalences in 2017.

Risk factor	Sex	Observed prevalence in 2017, % (CI)	Projected^a^ prevalence in 2017, % (CI)
Obesity	Men	23.6 (20.9, 26.2)	22.7 (19.2, 26.2)
	Women	21.2 (18.7, 23.8)	23.0 (19.8, 26.3)
Current smoking	Men	23.3 (20.8, 25.9)	22.2 (18.2, 26.2)
	Women	16.4 (14.2, 18.7)	13.7 (10.7, 16.8)
Hypertension	Men	42.1 (39.2, 45.0)	45.1 (41.5, 48.7)
	Women	27.1 (24.6, 29.5)	32.3 (29.1, 35.5)

a Prevalences projected using data from 1997 to 2012.

## Discussion

We presented a statistical method for producing prevalence projections of health indicators. We considered the future surveys as missing data which were multiply imputed by the chained equations technique. The imputed values were then combined as prevalence estimates. We were able to take into account the effects of background variables and their expected development in time. The method was demonstrated by projecting the prevalences of obesity, smoking and hypertension for the years 2020 and 2025.

The long-term increasing trend of obesity and decreasing trends of smoking and hypertension were projected to continue in the future. The results of hypertension demonstrated the importance of looking at the confidence intervals rather than the point estimates. Based on the past surveys, it may not be plausible that the prevalence among women would increase from 2017 to 2020, but on a large scale the projected trend seems believable.

The associations of the simulated risk factor values were checked to be consistent with the observations. We also evaluated the proposed method by comparing projections with the observed prevalences for the same year. However, this kind of comparison does not really prove the validity of the method, because if there is a substantial unexpected change in the trend, it could not be projected based on historical data. On the other hand, if the trend remains very steady, even a simplistic method could produce projections correctly on that straight line.

A possible application of our projection method could be to test different future scenarios. It could be investigated how fixing the values of some variables to the level of interest would change the projection of a health indicator. These kinds of scenario projections have been carried out using longitudinal data, for instance, in Härkänen et al. [18], in which the number of people with mobility limitations was projected. Instead of setting the predictor into a single value, a more realistic scenario could be tested by generating values of the predictor from a distribution possibly depending on some other background variables agreeing to the scenario.

It is important to note that when multiple imputation is used to predict future values, or other values outside the range of observations, the imputation model must be parametric. For example, although a non-parametric random forest would normally be a good choice for imputing missing values [19], it does not extrapolate properly beyond the range of observed values [20], such as for years in the future. Cautionary examples of implausible projections produced with random forest as the imputation method are presented in Supplemental Figures 4–6.

### Strengths and limitations

Ease of use is the strength of this method due to freely available flexible software for multiple imputation. It is straightforward to add explanatory variables into the imputation models. In addition, projections can be made for any year, that is, the ranges of projected time points are not restricted to survey intervals in observed data. Furthermore, no longitudinal data are needed to carry out the projection. On the other hand, longitudinal data could provide more reliable estimates on associations between variables and thus should be utilised if available.

A limitation of the proposed method is that it does not take into account the uncertainty related to population forecasts. A dynamic Bayesian model [21], for instance, could be developed to overcome this limitation, but at the same time, the easiness of application would be compromised. Another development could be to use resampling methods, such as bootstrapping [22], to take into account the sampling variability.

Projections are likely to be more or less dependent on the model used. We based our model selection on the Bayesian information criterion, but other criteria might lead to different models. One way to deal with the uncertainty related to the model selection would be using Bayesian model averaging [23]. This may, however, be sensitive to prior assumptions and thus would markedly complicate the application of the projection method. We recommend, however, carrying out sensitivity analyses with different specifications for the imputation models to get an idea of their effects on projections.

Population-level risk factor prevalences do not usually change rapidly, so these kinds of projections are typically needed with long ranges. What is long and short depends naturally on the context and the availability of data and survey time intervals. In principle, boundaries for how far the projections can be carried out using multiple imputation cannot be defined. However, it is important to assess and report the uncertainty of the projection as it tends to increase when the projection is carried out further in the future. As the projections are based on the assumption that the previously observed trends will continue, it should also be kept in mind that long-term projections are more prone to unexpected shocks than short-term projections [3].

## Conclusions

Using multiple imputation to simulate individual-level observations for the future based on observed cross-sectional survey data from the past can be used to calculate projections for health indicators. This was seen as a simple but effective method, which can take into account the anticipated changes in demography and other covariates. Sensitivity analyses were recommended to see the effect of the choice of the imputation models on projections.

## Supplemental Material

sj-docx-1-sjp-10.1177_14034948211061014 – Supplemental material for Projections for obesity, smoking and hypertension based on multiple imputation Click here for additional data file. Supplemental material, sj-docx-1-sjp-10.1177_14034948211061014 for Projections for obesity, smoking and hypertension based on multiple imputation by Jaakko Reinikainen, Tommi Härkänen and Hanna Tolonen in Scandinavian Journal of Public Health
