# Supplementary material for: Projections for obesity, smoking and hypertension based on multiple imputation
Source: Scand J Public Health. 2021 Dec 14;51(6):829–34. doi: 10.1177/14034948211061014 (PMC10350717; doi:10.1177/14034948211061014)
Supplement: sj-docx-1-sjp-10.1177_14034948211061014 – Supplemental material for Projections for obesity, smoking and hypertension based on multiple imputation [file sj-docx-1-sjp-10.1177_14034948211061014.docx]

**Supplement to “Projections for obesity, smoking and hypertension based on multiple imputation”**


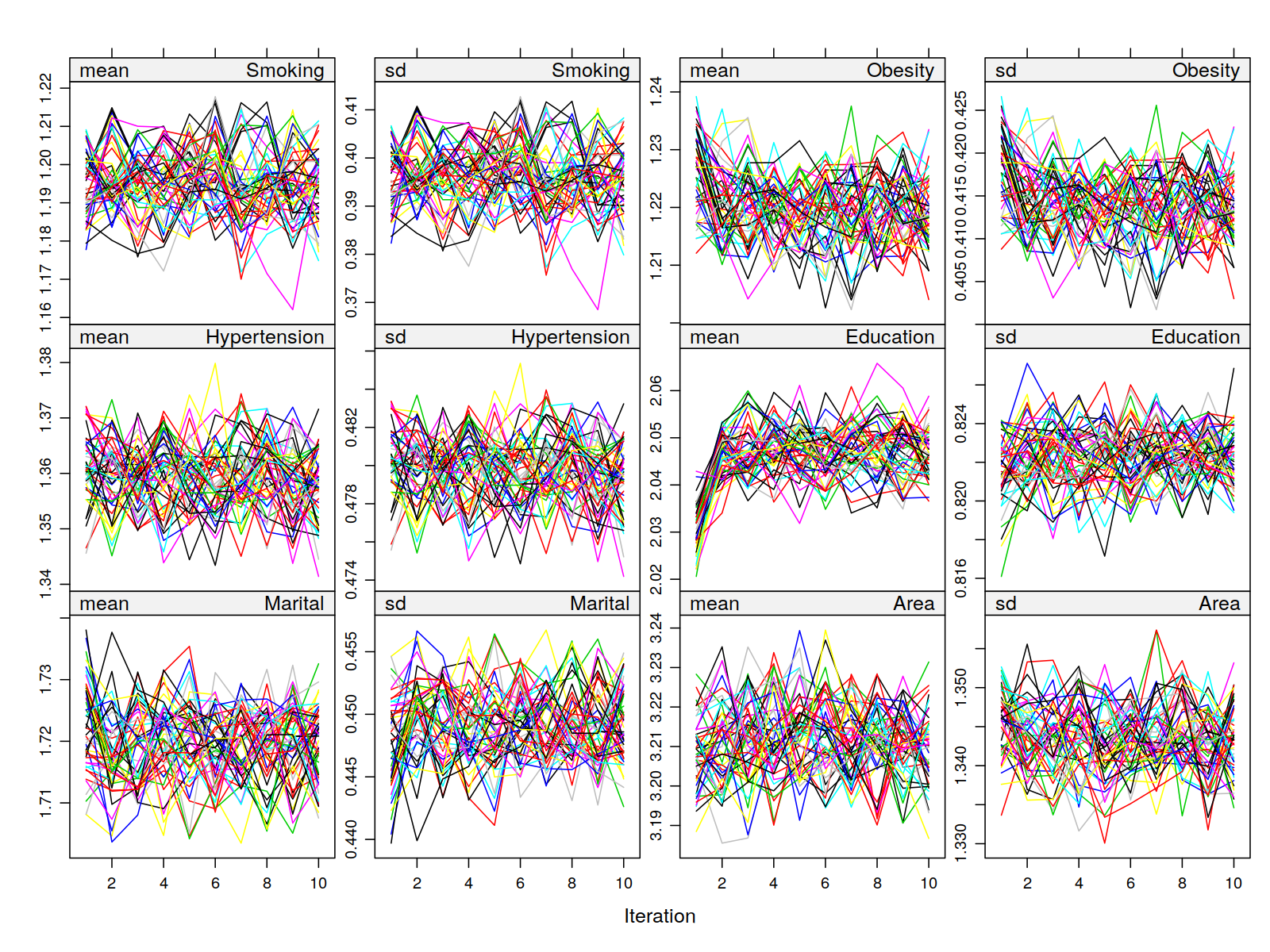


**Supplemental figure 1** Convergence plots showing means and standard deviations of the imputed values against the iteration number for each incomplete variable in 50 imputed data sets. The algorithm seems to have converged well, as the streams have mixed well without trends.


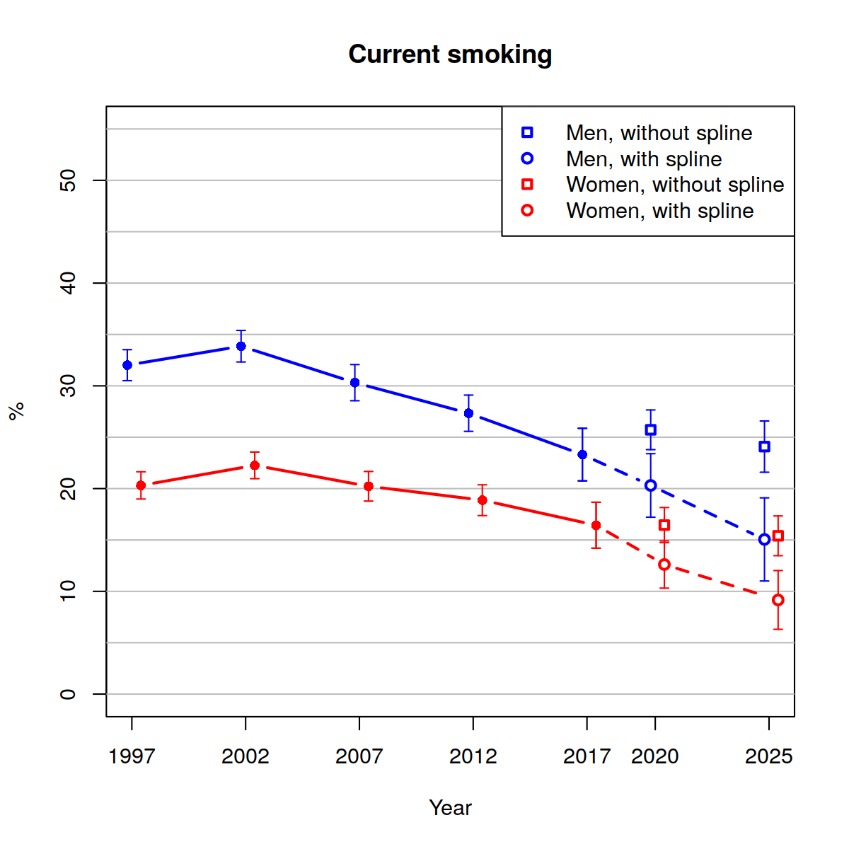


Supplemental figure 2 Comparison of projected prevalences of current smoking with and without restricted cubic splines for survey year in the imputation model.


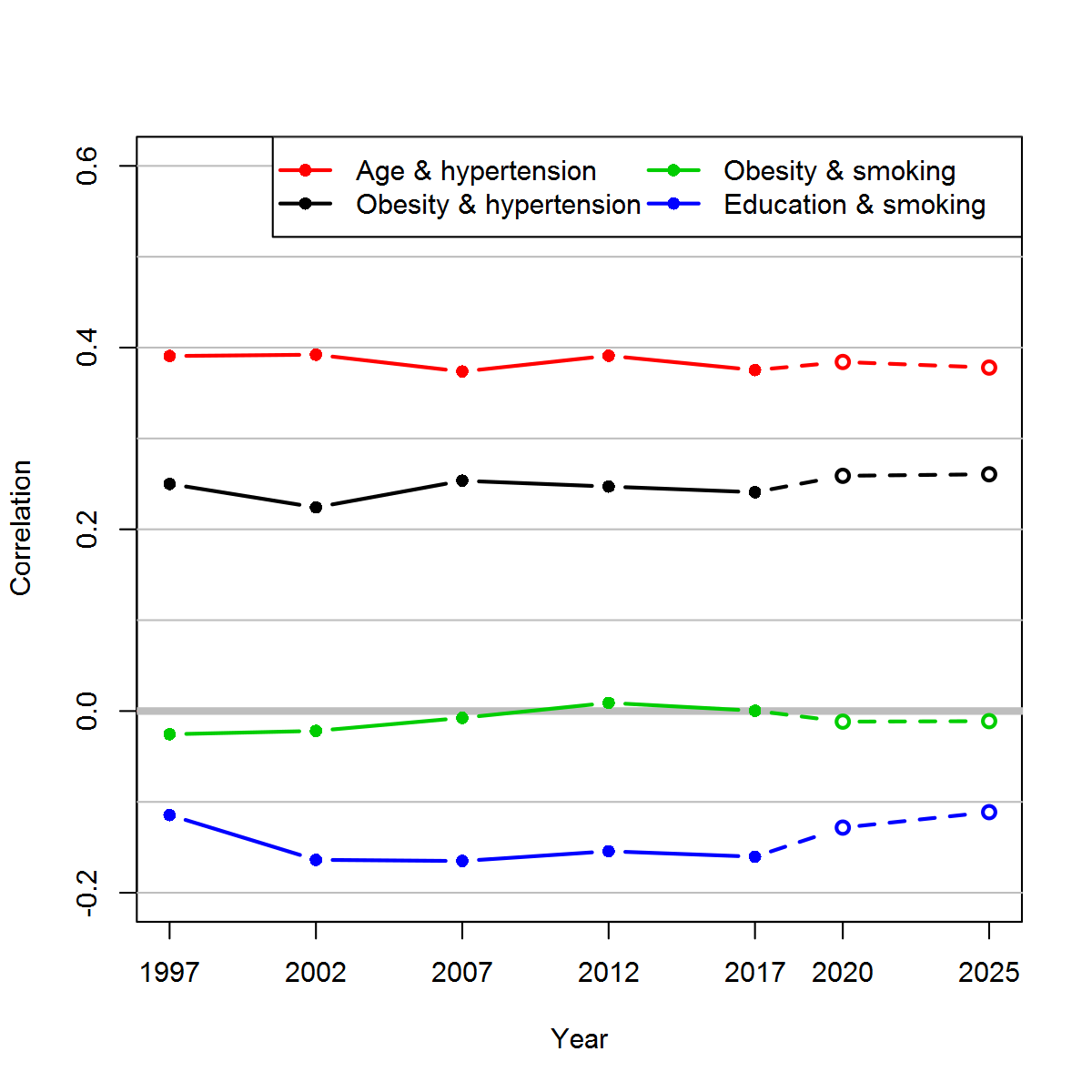


Supplemental figure 3 Examples of correlations in past surveys and in simulated future data.


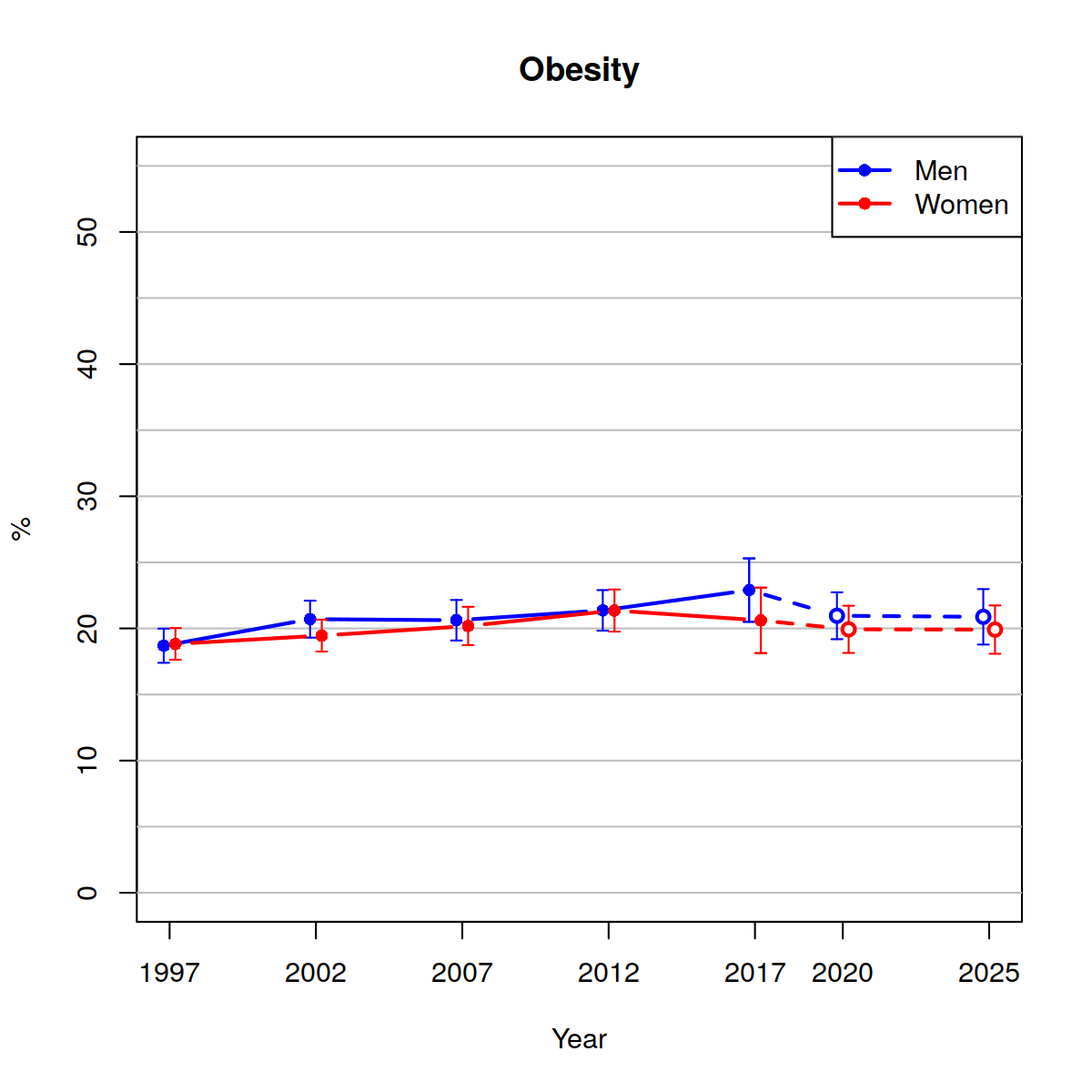


**Supplemental figure 4** Observed prevalences of obesity for the years 1997 – 2017 and projections for 2020 and 2025 produced using random forest as the imputation method.


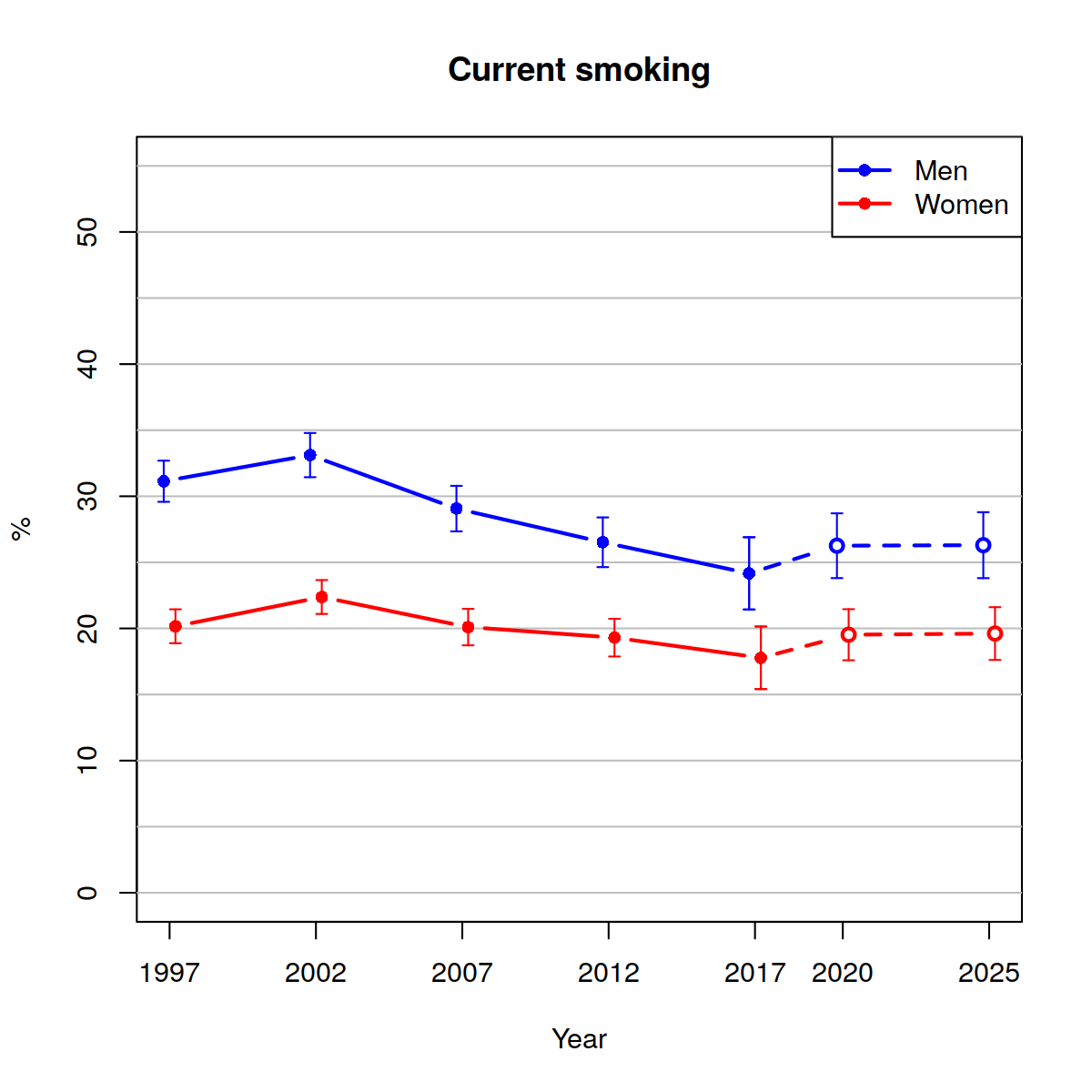


**Supplemental figure 5** Observed prevalences of current smoking for the years 1997 – 2017 and projections for 2020 and 2025 produced using random forest as the imputation method.


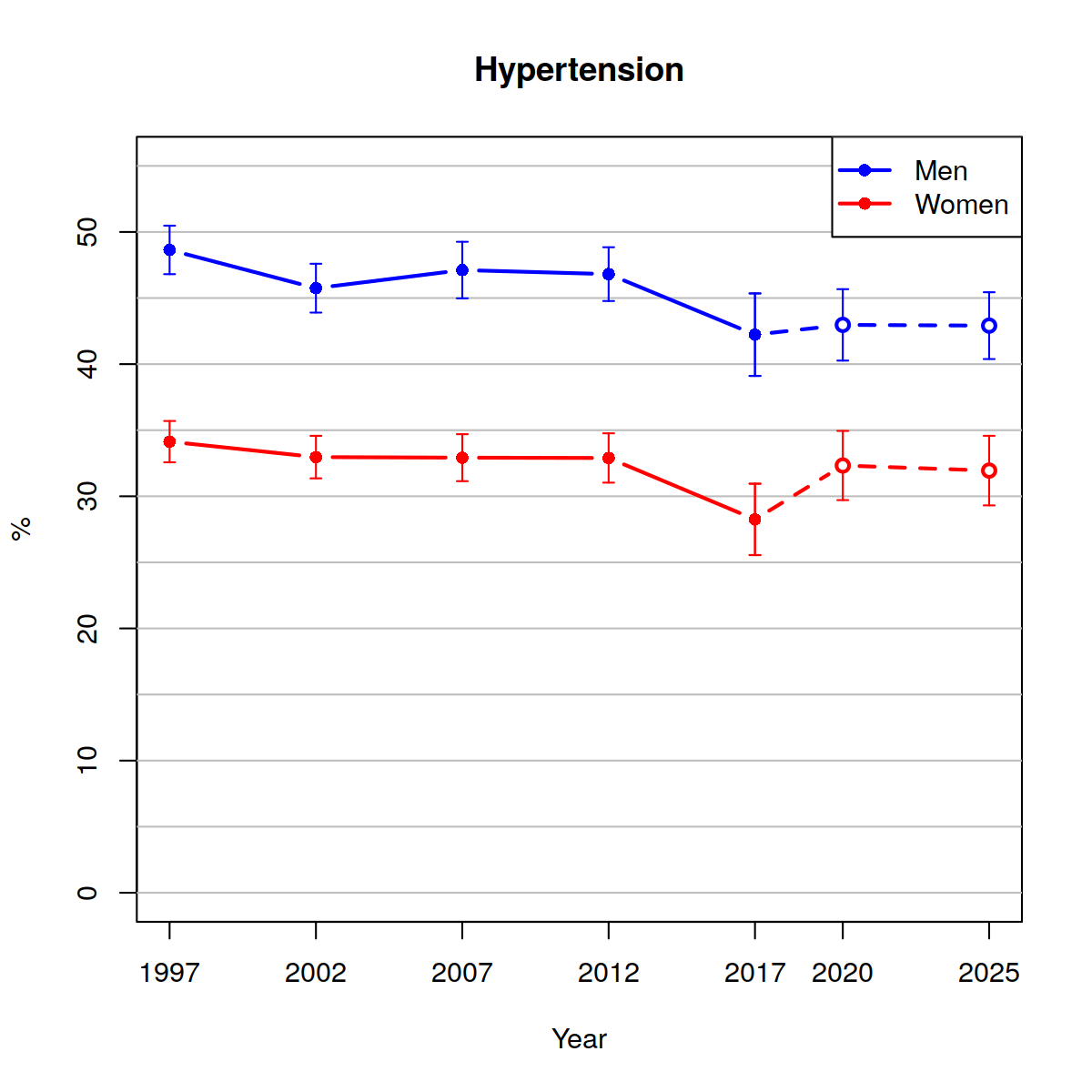


**Supplemental figure 6** Observed prevalences of hypertension for the years 1997 – 2017 and projections for 2020 and 2025 produced using random forest as the imputation method.
